# The ORIGINS Project: A Cross-Sectional Analysis of the Nutrition Profile of Pregnant Women in a Longitudinal Birth Cohort

**DOI:** 10.3390/nu16152571

**Published:** 2024-08-05

**Authors:** Poonam K. Pannu, Alexander J. J. Scherini, Desiree T. Silva, Sarah Whalan

**Affiliations:** 1 Telethon Kids Institute, Nedlands 6009, Australia; alex.scherini@telethonkids.org.au (A.J.J.S.); desiree.silva@telethonkids.org.au (D.T.S.); sarah.whalan@telethonkids.org.au (S.W.); 2 Faculty of Science, Medical School, University of Western Australia, Crawley 6009, Australia; 3 Joondalup Health Campus, Joondalup 6027, Australia

**Keywords:** ORIGINS, nutrition, pregnancy, cohort, DOHaD

## Abstract

Pregnancy is an opportunistic time for dietary intake to influence future disease susceptibility in offspring later in life. The ORIGINS Project was established to identify the factors that contribute to ‘a healthy start to life’ through a focus supporting childhood health and preventing disease (including non-communicable diseases). We aim to describe the dietary intakes of pregnant women in this cohort and to compare these to the Nutrient Reference Values (NRVs) and Australian Recommended Food Score (ARFS). The usual food and nutrient intakes of women were collected using the Australian Eating Survey (AES), a semi-quantitative food frequency questionnaire (FFQ). A total of 374 women completed the AES FFQ at both 20 weeks and 36 weeks of gestation between December 2016 and January 2023. Macronutrient, micronutrient, and food group intake were explored using descriptive statistics. Overall, it was found that the energy contribution from carbohydrates was low, while that from fat and saturated fat was high; participants were not meeting the recommendations for several key micronutrients (calcium, iron, iodine, and folate); and they had low diet quality scores for all food groups. These findings suggest that despite the ongoing promotion of healthy eating during pregnancy, further exploration into why dietary guidelines during pregnancy are not being adhered to is warranted.

## 1. Introduction

The epidemic of a range of chronic non-communicable diseases (NCDs) is a current worldwide challenge [1,2]. The majority of NCDs are preventable through the management of four behavioural risk factors: tobacco use, physical inactivity, harmful use of alcohol, and unhealthy diet [3,4]. In addition, NCDs involve a number of factors and result from complex interactions between individuals and the environment, such as vulnerability to risks [4]. Conversely, factors such as high socio-economic status and high levels of education and income may be conducive to a healthier dietary profile [5]. Early life can be a key time to institute preventative health measures [6]. The early environment during pregnancy is an opportunistic time to influence physiologic, metabolic, immune, and behavioural development which can affect future disease susceptibility [7].

The theory of the developmental origins of health and disease (DOHaD) suggests that the foetal origins of adult diseases are determined by perinatal exposure [8]. The maternal diet is an early exposure factor that can contribute to foetal development and induce long-term effects on the subsequent health of the child [9,10,11,12]. Studies have shown the link between a maternal diet that is high in processed food, characterized by low-nutrient, energy dense foods, and an increased risk of childhood over-growth and adiposity, along with the predisposition for cardiometabolic impairment during adulthood [7,13,14,15].

The maternal diet is also an influential factor in child health trajectory [9]. Nutrient deficiencies, such as in iron or iodine, have shown a link to impaired cognitive development in early childhood [16,17]. Dietary patterns that do not meet the recommended dietary guidelines during pregnancy have also been linked with asthma [18], dermatitis [19], and childhood obesity [14]. Conversely, a healthier diet during pregnancy may promote more favourable cognitive development in the child, including age-appropriate language abilities, reading and writing skills, and fewer affective problems in the child [17,20]. However, the study results are not conclusive; thus, further research is required in this area [21,22].

Longitudinal cohort studies provide rich data that allow researchers to investigate life course processes and to identify causal determinants of health and disease outcomes in later life [23]. Pregnancy studies are particularly crucial for exploring early origins of health and disease that begin in foetal life and infancy [6,7].

In the ORIGINS cohort of pregnant women, the aims of this study were to assess: (i) macronutrient intake; (ii) micronutrient intake, and (iii) maternal diet quality, comparing these results to the Nutrient Reference Values (NRVs) and the Australian Recommended Food Score (ARFS). There is limited data regarding dietary intakes in pregnant women in Australia; therefore, our study provides a meaningful overview of the nutrition intakes of these subjects.

## 2. Materials and Methods

### 2.1. Study Design and Participants

This cross-sectional study is nested within The ORIGINS Project, a collaboration between the Telethon Kids Institute and the Joondalup Health Campus (JHC). ORIGINS is one of the most comprehensive studies of pregnant women and their families in Australia to date, recruiting 10,000 families over a decade from the Joondalup and Wanneroo communities of Western Australia. The ORIGINS Project is an observational and interventional birth cohort study. Additional details of the cohort study protocol have been previously published [24].

Recruitment of participants for The ORIGINS Project took place at JHC. All pregnant women planning to deliver their babies at JHC, from both public and private hospitals, were eligible to enrol in the study at one of three timepoints during their pregnancies: 18 weeks, 28 weeks, and 36 weeks of gestation. Participants could enrol as an ‘active participant’ (*n* = 4016), which included invitations to complete in-depth self-reported surveys, clinic visits, sample collections, and to provide access to routine hospital and linked data, or as a ‘non-active participant’ (recruitment still ongoing), which includes access to routine hospital and linked data only. The participants included in this cross-sectional analysis come from the ‘active participant’ stream and include only those that provided dietary intake data at both 20 weeks and 36 weeks of gestation. Written informed consent was obtained from all study participants, whereby a member of The ORIGINS Project research team contacted the participants to ensure that each had a thorough understanding of the study.

### 2.2. Ethics

Ethics approval was granted by the Ramsay SA/WA Human Research Ethics Committee, Ref. 1440, 16 September 2016. The research related to human use complied with all the relevant national regulations and institutional policies, was performed in accordance with the tenets of the Helsinki Declaration, and was approved by the authors’ institutional review board or equivalent committee. Informed consent was obtained by the birthing mother antenatally, and she then provided consent on behalf of the child once it was born. The conducted research is not related to animal use.

### 2.3. Demographic Data

An online survey was completed by the active participants to obtain demographic data, including education level, ethnicity, employment history, marital status, and household income, along with information related to medical history and lifestyle factors following the participant’s enrolment into The ORIGINS Project.

### 2.4. Dietary Intake Data

The Australian Eating Survey (AES) [25], a validated Food Frequency Questionnaire (FFQ), was used to collect the participant’s usual food and nutrient intake over the previous 3–6 months. This online survey was sent to active participants at approximately 20 weeks gestation and then again at approximately 36 weeks gestation to ensure consistency in dietary intake between the two timepoints. The AES FFQ captures self-reported dietary intake using 120 semi-quantitative questions. Nutrient intakes from both dietary datasets have been quantified using the AUSNUT (Australian Food and Nutrient) 2011–13 database (Food Standards Australia New Zealand, Canberra, Australia) [26]. As part of the AES, participants are asked whether they take a supplement, but specific details about the supplement are not collected; therefore, our analysis is only based on food intake, not supplementation. 

### 2.5. Australian Recommended Food Score (ARFS)

The Australian Recommended Food Score (ARFS) is a brief food-based diet quality index that focuses on diet variety within the food groups recommended by the Australian Dietary Guidelines [27]. The ARFS is comprised of eight sub-sections comprising vegetables, fruit, meat/flesh foods, non-meat/flesh protein foods, breads and cereals, dairy foods, water, and spreads/sauces. Having a consumption frequency of ≥ once per week is allocated one point for most foods. ARFS is calculated by totalling the points for each food group, with the score ranging from 0 to 73 [28].

### 2.6. Nutrient Reference Values (NRVs)

Nutrient reference values (NRVs) have been developed by the National Health and Medical Research Council of Australia as a set of daily nutrient intake recommendations based on the current scientific knowledge [27]. To compare values to population level intakes, the estimated average requirement (EAR), adequate intake (AI), and acceptable macronutrient distribution range (AMDR) are the most appropriate references and are the focus of the current study [29,30]. The recommended dietary intake (RDI) has historically been used to assess population level nutritional intake; however, RDIs exceed the actual nutrient requirements of practically all healthy persons and are not synonymous with requirements [21]. Therefore, the Australian National Health and Medical Research Council recommends not using the RDI to assess the intakes of groups [29]. The estimated nutrient intakes for each participant were compared to these NRVs for the pregnancy life stage to determine whether the pregnant women in The ORIGINS Project were or were not meeting the recommendations.

### 2.7. Statistical Analysis

A descriptive cross-sectional analysis of the dietary intake data obtained from the cohort was undertaken. Only data from participants who had completed both the 20-week and 36-week gestation AES FFQ were included in the analysis. All data were tested for normality using the Shapiro–Wilk test for normality; those that were not normally distributed are presented as the median and interquartile range (IQR), or as a number and percentage, where appropriate. All statistical analyses were completed using STATA (16.1, StataCorp LLC, College Station, TX, USA).

Macronutrient consumption as a percentage of energy was calculated using the following formula:

$$\mathrm{Macronutrient}\;\mathrm{Energy}\;\%\;=\;\frac{\mathrm{Median}\;\mathrm{kJ}\;\mathrm{of}\;\mathrm{Macronutrient}\;\times \;\mathrm{Energy}\;\mathrm{Conversion}\;\mathrm{Factor}}{\mathrm{Median}\;\mathrm{Total}\;\mathrm{kJ}}$$

The energy conversion factors were 37.7 kJ/g for fats and 16.7 kJ/g for protein and carbohydrates (https://www.eatforhealth.gov.au/nutrient-reference-values/nutrients/dietary-energy, accessed on 5 March 2024).

## 3. Results

### 3.1. Participant Characteristics

Of the 3708 women recruited to the ORIGINS Project as active participants, 374, who completed both of the antenatal timepoints, were eligible for inclusion in this cross-sectional analysis. Participants completed the 20-week questionnaire between 15 and 29 weeks of gestation, while the 36-week questionnaire was completed between 32 and 40 weeks of gestation.

The baseline sociodemographic characteristics of the pregnant women from the ORIGINS Project who were included in this analysis are summarized in Table 1. The mean maternal age was 32.4 years (SD 4.4). Most women had completed a tertiary qualification (78%), were employed (78.1%), were married or in a de-facto relationship (82.1%), of a Caucasian background (82%), and had a combined household income of AUD 100,001 or more. The mean body mass index (BMI) of women pre-pregnancy was 26.2 (SD 6.1). Over 46% of the participants had a pre-pregnancy BMI that is considered overweight or obese. 

### 3.2. Macronutrient Intake

The macronutrient intake data is summarized in Table 2. Macronutrients that were explored comprise total fat (including saturated fat), protein, and carbohydrate (including sugar, starch, and fibre). The median energy intake of the pregnant women at 20 weeks and 36 weeks of gestation was 8169 kJ and 8209 kJ, respectively. The median intakes for total carbohydrate, protein, and fat remained consistent between 20 weeks and 36 weeks of gestation. Although carbohydrates were the largest source of energy for the women at both 20 weeks and 36 weeks of gestation, at 44.9% and 44.6% of total energy intake, respectively, this falls below the recommended range of 45–65% of total energy intake. The percentage of energy from protein was within the recommended ranges; however, total fat exceeded the recommended range of 20–35% of total energy intake, and saturated fat intake also exceeded the recommendation of <10% of total energy intake.

### 3.3. Micronutrient Intake

Micronutrients that were explored include: thiamine, riboflavin, niacin, vitamin C, folate, retinol, magnesium, phosphorous, calcium, iron, zinc, sodium, iodine, and potassium. Micronutrient intake data is displayed in Table 3. All micronutrients, excluding calcium, folate, iodine, and iron, met the EAR. Sodium and potassium exceeded the adequate intake (AI) recommendations.

### 3.4. Australian Recommended Food Score (ARFS)

The Australian Recommended Food Scores (ARFS) that were explored include: vegetables, fruit, meat/flesh foods, non-meat/flesh protein foods, breads and cereals, dairy foods, water, and spreads/sauces. Total ARFS was also included. ARFS data is displayed in Table 4, along with the maximum possible scores for each component. The total score at 20 weeks and 36 weeks of gestation was 35, out of a possible total score of 73.

## 4. Discussion

This study describes the food and nutrient intake of a population-based cohort of pregnant women and compares their intakes to recommendations based on the Nutrient Reference Values (NRVs) for the life stage of pregnancy, along with the Australian Recommended Food Score (ARFS). Overall, it was found that the macronutrient distribution of usual dietary intake fell within the recommendations for protein (within 15–25% of energy intake), fell below these recommendations for carbohydrate (outside 45–65% of energy intake), but exceeded the recommendations for total fat (exceeding the recommendation of 20–35% of energy intake) and saturated fat (exceeding the recommendation of <10% of energy intake). Many of the key micronutrient (calcium, iron, iodine and folate) intake targets for pregnancy were not being met. In addition, diet quality appeared to be poor, based on low ARFS for all food groups (total score of 35).

These findings are consistent with those of other Australian-based studies that have examined the dietary intake of pregnant women, indicating misalignment with current national healthy eating guidelines [22,31,32,33]. International studies have also found that pregnant women were not meeting the recommendations for iron [34] and iodine [35] in many European countries, and folate in the Netherlands population [36]. In 2011–12, the Australian Bureau of Statistics collected food and nutrient data as part of the Australian Health Survey [37]. This population-level survey reported that Australian women, aged 19–30 years, who were not pregnant had inadequate levels of calcium (71.3%), folate (10.9%), iron (37.5%), and iodine (11.7%) [37]. Furthermore, it was found that for these nutrients, which require an increased consumption during pregnancy, the pregnant women in our study did not meet the national recommendations, which has also been observed in similar studies [22,25,31,38]. Participants did not meet the EARs for calcium, folate, iodine, and iron. In a recent literature review of micronutrient intakes across pre-conception and pregnancy, sub-optimal intakes of iron, folate, and calcium relative to country-specific recommendations were found in Australia, Canada, China, Europe, India, Japan, and Pakistan [39]. Blumfield et al. [40] also reported similar findings in a systematic literature review of micronutrient intakes across pregnancy in developed countries. The dietary requirements for several nutrients, including folate, iodine, and iron, increase during pregnancy due to higher demand from the growing foetus; therefore, women often need to make changes to their diets or use supplements to meet the increased requirements [29].

This is of concern, as the antenatal period is a critical time for establishing risks of non-communicable diseases in offspring later in life [41]. It has been suggested that a higher quality diet during pregnancy is associated with a lower risk of gestational hypertension, as well as of lower birth weight [15,33], while a high fat, high sodium diet during pregnancy is associated with potential health consequences such as altered placental function and a pre-disposition to metabolic disease in the offspring [42].

Previous studies suggest that the dietary intake of pregnant women could be influenced by factors such as education level, socio-economic status, age, and pre-pregnancy BMI [5,43,44]. Results from similar Australian studies suggest that lower pre-pregnancy BMI and higher education levels are associated with higher vegetable intakes [39,43,45]. In contrast, low socio-economic groups generally have a more restrictive budget available for food purchasing, and fruit and vegetables can be overlooked [44]. The participants in this study had high levels of tertiary education, were employed, and of a Caucasian background; therefore, the assumption would be that their dietary intake and dietary quality would more closely align with the Australia dietary recommendations, which was not the case; thus, further exploration is required.

### 4.1. Calcium Intake

In our sample of pregnant women, calcium intake at both timepoints (789.9 mg and 827.6 mg, respectively) were slightly below the EAR of 840 mg. This is similar to the results of other studies that reported a median calcium intake as low as 695.3 mg [46], or slightly lower than other studies, which showed levels of 881 mg (16 weeks) and 958 mg (36 weeks) [47]. A systematic review of dietary calcium intake during pregnancy also found mean calcium intakes of 948.3 mg/day in high income countries, including Australia [48]. Adequate calcium intake is required during pregnancy for adequate bone turnover, and in preparation for lactation [49]. Low calcium intakes (≤738 mg/d) during pregnancy, in normotensive women, were found to correlate with an increased risk of hypertension after pregnancy [50]. However, calcium intakes (739–966 mg/d) closer to those in this study were determined to have no effect on hypertension after pregnancy [50]. Furthermore, calcium intake in the third trimester is predictive of bone mineral density in children at 16 years of age, showing the impact of the in-utero diet to beyond infancy [51]. Overall, it appears that calcium intake during pregnancy is below the recommend intakes, both in Australia and worldwide [38,46,50]. Based on these findings, a strategy is required to improve calcium intake during pregnancy [38,46,48,49,50,51].

### 4.2. Iron Intake

In our sample of pregnant women, iron intake at both timepoints (10.4 mg and 10.5 mg) was almost half the EAR of 22 mg. This is similar to another study based in Australia, which indicated intakes of 11.5 mg at both timepoints [47]. These lower iron intakes may indicate minimal iron supplementation during pregnancy, as comparable iron intakes from food were seen in another study (12.5 mg) [52], where supplement users had an additional 18.4 mg of iron. Iron deficiency is a global health concern, estimated to affect up to 52% of pregnant women across the world [53]. During pregnancy, iron supplementation is recommended to accommodate foetal and placental growth, increases in red cell mass, and for blood loss at delivery [52,54]. Lower intake of iron in early pregnancy may increase the risk of pre-term birth and low birth weight [54].

### 4.3. Iodine Intake

In our sample of pregnant women, iodine intake at both timepoints (122.3 µg and 126.3 µg) was below the EAR of 160 µg. This is similar to the results of other studies that have reported low iodine intake [22,46]. Maneschi et al. [22] also reported 119 µg in an early pregnancy cohort and 130 µg in a late pregnancy cohort. Iodine has been found to reduce the risk of neurological development issues in the growing child [55,56]. Zhou et al. [55] found that a maternal iodine intake of less than 220 µg/day or more than 391 µg/day was associated with poorer cognitive, language, and motor scores in child.

### 4.4. Folate Intake

In our sample of pregnant women, folate intake at both timepoints (309.4 µg and 313.8 µg) was almost half the EAR of 520 µg. This was a lower intake compared to that of 563.9 µg reported in another study [57]. Maneschi et al. [22] also reported higher intakes of 521 µg and 541 µg in early and late pregnancy, respectively. Adequate folate intake has been shown to reduce the occurrence of neural tube defects, such as spina bifida and anencephaly in neonates [58]. Folate deficiency during pregnancy can also lead to increased risk of adverse outcomes like pre-eclampsia and foetal anomalies [59]. 

### 4.5. Sodium Intake

During pregnancy, women were found to exceed the AI for sodium. The AI is set generously at 460–920 mg; however, women at 18 weeks and 36 weeks of gestation were consuming more than double the AI (1823.6 mg and 1856.2 mg, respectively.) This is consistent with other studies, where the AI for sodium appears to be exceeded [47]. Sodium intake during pregnancy is important for regulation of maternal blood pressure and maternal blood circulation in the placenta [60]. There is limited evidence on restriction of sodium intake and reduction in gestational hypertension and development of preeclampsia [61,62]. In addition, as there is no upper limit set for sodium intake during pregnancy or in adulthood, further research is required to determine the most reasonable limit.

### 4.6. Australian Recommended Food Score (ARFS)

The ARFS is used to indicate relative adherence to the Australian Guide to Healthy Eating (AGHE) recommendations, with higher scores indicate higher diet quality and alignment with AGHE recommendations for adults [25]. A score above 47 points is classified as “outstanding”; a score of 39–46 points is “excellent”; a score of 33–38 is “getting there”; and a score below 33 “needs work” [45]. The ARFS score of 35 at both 20 weeks and 36 weeks of gestation in our study participants indicate a “getting there” result, falling much lower than the maximum possible score of 73. Similar scores, which appeared to improve slightly during pregnancy, were found in the pilot BABY1000 study, with median ARFS of 37 in preconception/early pregnancy and 38 in late pregnancy [22]. Similar scores were also found in a sample from the Australian Longitudinal Study on Women’s Health [63], which obtained a total score of 32, and a study undertaken by Harper et al. [46], which found a total ARFS score of 31 in a cohort of pregnant women with gestational diabetes.

### 4.7. Strengths and Limitations

There is limited data on dietary intakes in pregnant women in Australia [22,47,63]. Thus, our study provides a meaningful overview of the nutrient intakes of pregnant women in this country. However, the ORIGINS cohort cannot be regarded as a representative sample of pregnant women in Australia. The women who had completed both the 20-week and 36-week gestation FFQ had high levels of tertiary education, were mostly of Caucasian backgrounds, and the majority were employed. This demographic profile was not surprising, as there is often an issue of self-selection bias with nutrition-based research surveys [63]. Despite higher SES and education attainment, this sample is still fails to meet the dietary guidelines. However, our results are comparable to those of other studies of dietary intakes during pregnancy [22,25,31,38], thus providing evidence for future studies. 

The AES FFQ is an established tool which is widely used in dietary intake studies [25]. Therefore, we are able to compare our results to those of other cohort studies using a similar tool. In addition, the ARFS, which is a new diet quality scoring system designed to align with the Australian Dietary Guidelines, has been found to be a suitable tool to evaluate dietary quality and nutrient intakes in adults [27].

Longitudinal cohort studies allow for repeated measures and surveys. Though non-repeated FFQs can reduce subject burden, they may not capture the changes to macronutrient and micronutrient intake across the course of pregnancy [43]. Repeated FFQs can account for seasonal changes in food group intakes [33]; they can also help to capture the impacts of morning sickness and satiety during the different trimesters of pregnancy [43]. We included only participants who had completed both the 20-week and 36-week gestation timepoints in this analysis, which reduced the number of participants substantially, as we found that the women completed one or the other of the antenatal Australian Eating Survey. Unfortunately, this survey placed a high burden on participants, which impacted compliance.

Data is often not collected for supplementation in maternal diets [31,32,38,43,64]. The AES FFQ collects information regarding nutritional supplementation; however it is reliant on self-reporting, and it was not possible to determine the micronutrient contribution of supplementation due to a lack of detail on the brand and dosage used [31]. Approximately 49% of participants reported supplement use at 20 weeks of gestation, which increased to 54% at 36 weeks. For the context of this study, the aim was to determine whether pregnant women are meeting the NRV and ARFS targets from food intake alone. This provides a useful benchmark for the nutritional adequacy and dietary intakes of pregnant women in this cohort.

The findings from this study are important for advising dietary recommendations for pregnancy and the development of the child, both antenatally and postnatally. The findings provide a strong basis for designing epidemiological studies within the cohort of pregnant women, where there are increasing rates of metabolic diseases, such as type 2 diabetes and cardiovascular diseases. The fact that pregnant women in this cohort were consuming adequate intakes of macronutrients, but not receiving enough of the essential micronutrients, needs to be further explored, especially considering how these nutrients impact health outcomes for both the mother and their offspring. The next steps include exploring the association between maternal dietary intakes and birth outcomes, such as infant weight and body composition at 1, 3, and 5 years of age.

## 5. Conclusions

Overall, it was found that the pregnant women in this study had a macronutrient distribution that was relatively high in total fat and saturated fat and low in carbohydrates, and many of the key micronutrient intake targets for pregnancy were not being met for calcium, iron, iodine, and folate. In addition, diet quality appeared to be poor, based on low ARFS scores for all food groups. These findings suggest that despite ongoing promotion of healthy eating during pregnancy, more support is required to assist pregnant women in achieving optimal dietary intake, not only for themselves, but also for the long-term health of their offspring.

## Figures and Tables

**Table 1:** Sociodemographic characteristics of pregnant women in the ORIGINS Project (*n* = 374).

Variables	Value	
	Mean	SD
Maternal Age (years)	32.4	4.4
Height (cm)	165.9	14.4
Weight (kg)	72.8	14.4
Parity	1.2	1.4
Number of adults 18 and over in household	2.1	0.4
	Number (%)	
BMI (pre-pregnancy)		
<18.5	6 (1.6)	
18.5–24.99	141 (37.7)	
25–29.99	110 (29.4)	
≥30	65 (17.4)	
Missing	52	
Cultural Background		
Caucasian	309 (82.6)	
Aboriginal or Torres Strait Islander Descent	5 (1.3)	
Asian	25 (6.7)	
African	6 (1.6)	
Not Specified	34 (9.1)	
Born in Australia	224 (59.9)	
Income		
Up to AUD 25,000	8 (2.14)	
AUD 25,001–AUD 50,000	6 (1.6)	
AUD 50,001–AUD 75,000	26 (7)	
AUD 75,001–AUD 100,000	46 (12.3)	
AUD 100,001–AUD 150,000	129 (34.5)	
More than AUD 150,000	122 (32.6)	
Missing	37	
Education		
High School or Lower	61 (16.3)	
Tertiary Qualification	292 (78.1)	
Missing	21	
Marital Status		
Married/De Facto	307 (82.1)	
Not Married	15 (4.0)	
Missing	52	
Employment		
Employed	292 (78.1)	
Not Employed	56 (15.0)	
Missing	26	

**Table 2:** Estimated macronutrient intakes of pregnant women in the ORIGINS Project (*n* = 374).

Nutrients	Median		IQR		% of Energy		Recommendations * (% Energy)
	20 Weeks	36 Weeks	20 Weeks	36 Weeks	20 Weeks	36 Weeks	
Energy (kJ)	8169	8209	2684.5	3021.8	-	-	-
Protein (g)	86.2	88.2	32.7	34.6	17.6	17.7	15–25
Total Fat (g)	**78.5 **	**81.9**	30.3	32.5	**36.9 **	**37.3**	**20–35**
Saturated Fat (g)	**30.1 **	**31.7**	12.3	14.0	**14.2**	**14.5**	**<10**
Total Carbohydrate (g)	217.6	219	88.4	87.9	**44.9 **	**44.6**	**45–65**
Sugar (g)	103.0	107.2	48.7	53.1	-	-	10–25
Starch (g)	111.8	109.4	52.3	51.6	-	-	
Fibre (g)	26.0	25.9	11.1	10.4	-	-	

* Note: Bolded nutrients did not meet the recommendations. Adapted from Nutrient Reference Values for Australia and New Zealand, including Recommended Dietary Intakes (p. 252), by NHMRC, the Department of Health and Aged Care [DHaA], and NZMH, 2006, (https://www.nhmrc.gov.au/about-us/publications/nutrient-reference-values-australia-and-new-zealand-including-recommended-dietary-intakes, accessed on 5 March 2024). Copyright 2016 by Commonwealth of Australia.

**Table 3:** Estimated micronutrient intakes of pregnant women in the ORIGINS Project (*n* = 374).

Nutrients	Median		IQR		EAR *
	20-Weeks	36-Weeks	20-Weeks	36-Weeks	
Thiamine (mg)	1.5	1.5	0.8	0.7	1.2
Riboflavin (mg)	1.9	2.03	1.0	1.0	1.2
Niacin Equivalents (mg)	36.6	38.1	13.6	13.3	14
Vitamin C (mg)	149.1	144	91.5	98.4	40
Folate (µg)	**309.4**	**313.8**	135.5	141.2	**520**
Retinol Equivalents (µg)	807.1	822.5	492.2	482.0	550
Magnesium (mg)	370.0	375.7	124.3	125.8	290–300 **
Phosphorus (mg)	1358.0	1401.9	518.7	596.3	580
Calcium (mg)	**789.9**	**827.6**	403.4	454.9	**1000**
Iron (mg)	**10.4**	**10.5**	3.8	4.3	**22**
Zinc (mg)	10.9	11.1	2.0	2.3	9
Sodium (mg)	**1823.6**	**1856.2**	817.1	822.6	460–920 ***
Iodine (µg)	**122.3**	**126.3**	58.4	66.6	**160**
Potassium (mg)	3065.6	3132.3	1052.3	1233.4	2800 ***

* Note: bolded nutrients did not meet the recommendations. EAR, Estimated Average Requirement for pregnant women aged 19–50 years. Adapted from Nutrient Reference Values for Australia and New Zealand, including Recommended Dietary Intakes (pp. 283, 285, 287, 289, 291), by the NHMRC, Department of Health and Aged Care [DHaA], and NZMH, 2006, (https://www.nhmrc.gov.au/about-us/publications/nutrient-reference-values-australia-and-new-zealand-including-recommended-dietary-intakes, accessed on 5 March 2024). Copyright 2016 by Commonwealth of Australia. ** Magnesium RDI for 19–30, 350 mg; 31–59 y, 360 mg. *** Sodium and potassium show Adequate Intake (AI) (AI, adequate intake for pregnant women aged 19–50).

**Table 4:** Australian Recommended Food Scores (ARFS) for pregnant women in the ORIGINS Project (*n* = 374).

Food Groups	Median		IQR		Maximum Score *
	20 Weeks	36 Weeks	20 Weeks	36 Weeks	
Vegetables	13	13	7	6	21
Fruit	6	6	4	4	12
Breads and cereals	6	6	3	2.75	13
Meat/Flesh foods	2	2	1	1	7
Non-Meat/Flesh protein foods	2	2	1	1	6
Dairy foods	4	4	2	3	11
Water	1	1	0	0	1
Spreads/Sauces	1	1	2	2	2
Total Score	35	35	13	13	73

* Note. Maximum Score values adapted from “The Comparative Validity and Reproducibility of a Diet Quality Index for Adults: The Australian Recommended Food Score” [28]. Copyright 2015 by the authors.

## Data Availability

The data presented in this study are available on request from the corresponding author due to privacy reasons.
